# Impact of fluvoxamine on outpatient treatment of COVID-19 in Honduras in a prospective observational real-world study

**DOI:** 10.3389/fphar.2022.1054644

**Published:** 2022-11-30

**Authors:** Estela Pineda, Jarmanjeet Singh, Miguel Vargas Pineda, Jose Garay Umanzor, Fernando Baires, Luis G. Benitez, Cesar Burgos, Anupamjeet Kaur Sekhon, Nicole Crisp, Anita S. Lewis, Jana Radwanski, Marco Bermudez, Karen Sanchez Barjun, Oscar Diaz, Elsa Palou, Rossany E. Escalante, Carlos Isai Hernandez, Mark L. Stevens, Deke Eberhard, Manuel Sierra, Tito Alvarado, Omar Videa, Miguel Sierra-Hoffman, Fernando Valerio-Pascua

**Affiliations:** ^1^ Department of Internal Medicine Hospital CEMESA, San Pedro Sula, Honduras; ^2^ Department of Cardiovascular Medicine, University of California, Riverside, Riverside, CA, United States; ^3^ Department of Internal Medicine Hospital Mario Catarino Rivas, San Pedro Sula, Honduras; ^4^ Department of Obstetrics and Gynecology Hospital Mario Catarino Rivas, San Pedro Sula, Honduras; ^5^ Universidad Nacional Autónoma de Honduras, Tegucigalpa, Honduras; ^6^ Sleep Medicine, Kaiser Permanente, Fontana, CA, United States; ^7^ Wound Care Department El Campo Memorial Hospital, El Campo, TX, United States; ^8^ Pharmacy Department El Campo Memorial Hospital, El Campo, TX, United States; ^9^ Pharmacy Department Citizens Hospital, Victoria, TX, United States; ^10^ Department of Medicine SBH Health System, Bronx, NY, United States; ^11^ Department of Critical Care Hospital Regional del Norte Instituto Hondureño de Seguridad Social, San Pedro Sula, Honduras; ^12^ Internal Medicine Department, Facultad de Ciencas Médicas, Universidad Nacional Autónoma de Honduras, Tegucigalpa, Honduras; ^13^ Department of Medicine, Facultad de Ciencas Médicas, Universidad Nacional Autónoma de Honduras, Tegucigalpa, Honduras; ^14^ HEME Clinic, Choluteca, Honduras; ^15^ Research Department, Texas A&M College of Medicine, Detar Family Medicine Residency Program, Victoria, TX, United States; ^16^ Universidad Tecnológica Centroamericana, Tegucigalpa, Honduras; ^17^ Infectiology Department, Facultad de Ciencias Médicas, Universidad Nacional Autónoma de Honduras, Tegucigalpa, Honduras; ^18^ Clínica de Atención Medica Integral CAMI, Tegucigalpa, Honduras; ^19^ Research and Infectious Disease Department, Texas A&M College of Medicine, Detar Family Medicine Residency Program, Victoria, TX, United States; ^20^ Department of Critical Care Hospital CEMESA, San Pedro Sula, Honduras

**Keywords:** fluvoxamine, COVID-19, repurposed drugs, early outpatient treatment, Honduras

## Abstract

**Background:** The COVID-19 pandemic has impacted millions of lives globally. While COVID-19 did not discriminate against developed or developing nations, it has been a significant challenge for third world countries like Honduras to have widespread availability of advanced therapies. The concept of early treatment was almost unheard of when early outpatient treatments utilizing repurposed drugs in Latin American countries began showing promising results. One such drug is fluvoxamine, which has shown tremendous potential in two major studies. As a direct result, fluvoxamine was added to the standard of care in a major medical center outpatient COVID-19 clinic.

**Methods:** This is a prospective observational study performed at the Hospital Centro Médico Sampedrano (CEMESA) in San Pedro Sula, Cortes, Honduras in the COVID-19 outpatient clinic. All patients were at least 15 years of age who had presented with mild or moderate signs and symptoms of COVID-19, and who also had a documented positive SARS-CoV-2 antigen or Reverse Transcription Polymerase Chain Reaction (RT-PCR) were included in the study. These patients then were all prescribed fluvoxamine. The cohort of patients who decided to take fluvoxamine were compared for primary endpoints of mortality and hospitalization risk to the cohort who did not take fluvoxamine. Patients were then monitored for 30 days with the first follow up at 7 days and the second follow up at 10–14 days of symptom onset. Categorical variables were compared by Pearson Chi-square test. The Relative risk was calculated using regression models. Continuous variables were compared by t-test and Wilcoxon rank-sum tests.

**Results:** Out of total 657 COVID-19 cases, 594 patients took fluvoxamine and 63 did not take fluvoxamine. A total of five patients (0.76 percent) died, with only one death occurring in the fluvoxamine group. Patients who received fluvoxamine had a significantly lower relative risk of mortality (RR 0.06, *p* 0.011, 95% CI 0.007–0.516). There was a lower relative risk of hospitalization in the patients who in the fluvoxamine group. (−10 vs. 30 hospitalizations, RR 0.49, *p* = 0.035, 95% CI 0.26–0.95). There was 73 percent reduction in relative risk of requiring oxygen in the fluvoxamine group (RR 0.27, *p* < 0.001, 95% CI 0.14–0.54 Mean lymphocytes count on the first follow-up visit was significantly higher in the fluvoxamine group (1.72 vs. 1.38, Δ 0.33, *p* 0.007, CI 0.09–0.58).

**Conclusion:** The results of our study suggest that fluvoxamine lowers the relative risk of death, hospitalization, and oxygen requirement in COVID 19 patients.

## 1 Introduction

The coronavirus disease 2019 (COVID-19) pandemic that originated in Wuhan, China remains not only a topic of heated debate, but also poses a threat to our healthcare systems, society, and overall economy. For most of 2020 and part of 2021, skepticism, doubt, and fear of the unknown could be globally palpated. The concept of early treatment was almost unheard of with most international scientific bodies only recommending isolating at home and symptomatic treatment. The lack of consistent therapeutic guidelines left many patients anxiously waiting until they developed severe disease before a healthcare provider would assess their condition. At this point, it was often too late to prevent them from succumbing to the virus.

As early as January 2020, the clinical evidence became clear. Patients with Severe Acute Respiratory Syndrome Coronavirus 2 (SARS-CoV-2) demonstrated an identifiable pattern. Survivors typically were young and showed very low or minimally activated inflammatory markers and pro-thrombotic state. In contrast, non-survivors had quite the opposite profile with hyperinflammatory and pro-coagulant immunological profiles (Wang et al., 2020).

Unlike developed countries, countries in development did not have the luxury of waiting for 1A evidence regarding potential treatments. Instead, early treatment protocols with repurposed drugs were developed.

Repurposed drugs have been commonplace throughout the history of medicine. These are drugs that were originally studied for a specific pathology; however, inadvertently found to work for distinctly different disease processes. Some well-known examples include sildenafil, hydroxychloroquine, ivermectin, colchicine, tocilizumab, and metformin. They display anti-inflammatory properties that could be of potential use in ameliorating the hyperinflammatory phases of the disease (Meikle et al., 2021), and improving the clinically related COVID-19 symptomatology (Alsabhan and Alshammari, 2022). These medications have been in use for more than 30 years, are deemed safe, and have established side effect profile. They are also readily available (Facente et al., 2021). Thus, medicine must always leave the door open to new discoveries and avenues for medications formulated years ago. Time and time again, research has shown the importance of repurposed drugs. Those discoveries are the essence of medicine. However, most of the developed world took an opposite approach, by focusing on COVID-19 specific pharmaceuticals. Honduras lacked the financial means to implement or manufacture novel drugs, thus, compelling the use of the best available evidence in that moment, even when opposing results emerged (Bhuta et al., 2022; Ontai et al., 2022; Valerio Pascua et al., 2022).

Even though Honduras is a low to middle income Central American country, with an extremely underserved and understaffed medical system, the government proactively recommended early outpatient treatment of COVID-19 to avoid collapse of their healthcare system. By the spring of 2020, based on the available evidence, safe repurposed drugs were already being used in an established protocol. Only 1 week after initiation of this national protocol, a significant reduction of the case fatality rate was noted (Valerio Pascua et al., 2021; Ontai et al., 2022; Secretaria de Salud Honduras, 2020). After the publication of a randomized control study by Lenze et al. that showed prevention of clinical deterioration in the early treatment for COVID-19 with fluvoxamine use, (Lenze et al., 2020), it was offered as a treatment option in addition to the standard of care protocol in Honduras (Valerio Pascua et al., 2022). Here we present a real-world observational study done in Honduras, a nation in development, to evaluate the effectiveness of fluvoxamine in preventing clinical deterioration in terms of mortality and hospitalization.

## 2 Methods

We conducted this prospective observational study with data collected from medical records from November 2020 until January 2022. The study was approved by the Institutional Review Board (IRB) of the Hospital Centro Médico Sampedrano and the Ethics committee of investigation of infectious and zoonotic disease masters of the Universidad Nacional Autónoma de Honduras. This study was performed in the COVID-19 outpatient clinic at the Hospital Centro Médico Sampedrano (CEMESA) in San Pedro Sula, Cortes, Honduras. Patients at least 15 years of age with mild to moderate COVID-19 signs and symptoms, and a positive SARS-CoV-2 antigen or Reverse Transcription Polymerase Chain Reaction (RT-PCR) were included in the study. Individuals who developed signs and symptoms of the disease that had been in close contact with one of the patients in the cohort were also treated and included in the study. Pregnant women, patients younger than 15 years of age, and patients with severe and critical COVID-19 presentation were excluded.

Fluvoxamine was prescribed as part of the early treatment to all patients presenting with mild to moderate COVID-19 disease. While fluvoxamine was recommended to everyone, a cohort of patients chose not to take fluvoxamine and became the control group. All patients were monitored for 30 days and had a follow up evaluation at 7 and 10–14 days after the onset of symptoms. Patients were followed using telemedicine if they had new concerning symptoms such shortness of breath, chest pain, or oxygen desaturation. Patients were started on fluvoxamine 50 mg orally twice daily for 3 days and titrated up to 100 mg two or three times a day depending on patient tolerance and disease severity to complete a 14-day course (Valerio Pascua et al., 2022).

Mild Disease: Patient’s that presented with any of the common signs or symptoms of SARS-CoV-2 infection, including fever, cough, sore throat, malaise, headache, myalgias, nausea, vomiting, diarrhea or loss of taste and smell were included in this group. They were excluded if they had dyspnea, hypoxia, or abnormal chest radiography.

Moderate Disease: Patients who demonstrated evidence of lower respiratory disease (e.g., dyspnea) at clinical presentation, and/or abnormal chest radiography, and had a pulse oximeter saturation of greater than 94% on room air were included in this group.

Severe Disease: Patients who demonstrated evidence of lower respiratory disease with a pulse oximetry of less than 94% on room air, a ratio of arterial partial pressure of oxygen to fraction of inspired oxygen (PaO2/FiO2) of less than 300 mmHg, a respiratory rate of greater than 30 breaths per min, and/or an abnormal chest radiography demonstrating greater 50% pulmonary infiltrates were included in this group.

Critical Illness: This group was defined as individuals presenting with respiratory failure, septic shock, and/or multiple organ dysfunction.

The primary endpoints of the study included outcomes of 30-day mortality and hospitalization. 30-day mortality included any event of death after index visit from any cause other than homicide or accidental death. Hospitalization included all patients who were hospitalized within 30 days of index visit and due to COVID-related prodromal symptoms. Secondary outcomes of the study included oxygen requirement within 30 days of index visit, tocilizumab requirement within 30 days after index visit, duration of hospital stay (days in the hospital), and progression of disease from mild/moderate category to severe/critical category. We also measured laboratory markers including differences in lymphocyte count and inflammatory markers like CRP, ESR, Procalcitonin etc.

### 2.1 Statistical analysis

Statistical analysis was performed using Stata 17.0 Basic edition software. Baseline descriptive statistics were performed on all variables. Inferential statistics were conducted *via* Pearson Chi-square testing for categorical data outcomes. The relative risk ratio (RR) was calculated using generalized linear model regression. Univariate analysis was used to check for significant differences in baseline characteristics between fluvoxamine and non-fluvoxamine groups. Propensity Score matching could not be statistically performed due to near absence of vaccinated patients in the non-fluvoxamine group and due to low number of overall events. However, considering the possibility of confounding from lack of vaccinated patients in the non-fluvoxamine group, we performed analysis excluding vaccinated patients. Continuous variables were compared by t-test and Wilcoxon rank-sum tests. The Wilcoxon rank-sum test was used primarily for non-parsimonious continuous data variables.

## 3 Results

**FIGURE 1:**
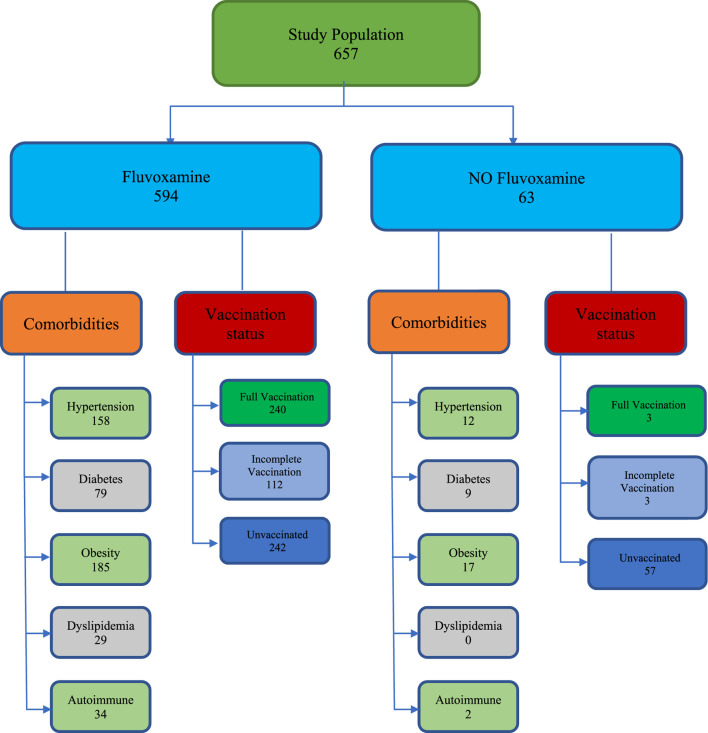
Patient selection criteria.

Of 657 total patients in the study, 330 were males (50.2%). A total of 594 patients took fluvoxamine, and 63 patients did not take fluvoxamine (Figure 1). The mean age of the fluvoxamine group was 47.9 years, and the non-fluvoxamine group was 49.7 years (*p* value 0.409). A total of 170 patients had hypertension (158 in fluvoxamine group vs. 12 in non-fluvoxamine group, *p* 0.218), 88 had diabetes (79 in fluvoxamine group vs. 9 in non-fluvoxamine group, *p* 0.785), and 202 were obese (185 in fluvoxamine group vs. 17 in non-fluvoxamine group, *p* 0.697). 243 patients were un-vaccinated in the Fluvoxamine group and 57 were unvaccinated in the non-fluvoxamine group. Eleven patients were started on steroids prior to their index visit (by outside providers). There was no significant difference between prior steroid use between both groups (*p* = 0.276). All other baseline parameters as shown in Table 1 were similar between the groups.

**TABLE 1:** Baseline characteristics of the study cohort according to fluvoxamine use.

Characteristic	Cohort participants	Received fluvoxamine	Not- received fluvoxamine	*p* value
	No (Percent)	Number (Percent)	Number (Percent)	
Total	657	594 (90.6%)	63 (9.4%)	
Sex				0.761
Female	327 (49.8%)	295 (49.7%)	32 (50.8%)	
Male	330 (50.2%)	300 (50.5%)	30 (47.6%)	
Mean Age	48.1 years	47.9 years	49.7 years	0.409
Comorbidities				
Hypertension	170 (25.8%)	158 (26.6%)	12 (19.0%)	0.218
Diabetes	88 (13.4%)	79 (13.3%)	9 (14.2%)	0.785
Obesity	202 (32.7%)	185 (31%)	17 (27%)	0.697
Dyslipidemia	29 (4.4%)	29 (4.8%)	0 (0.0%)	0.075
Autoimmune	36 (5.5%)	34 (5.7%)	2 (3.2%)	0.413
Heart disease	11 (1.7%)	11 (1.7%)	0 (0%)	0.276
Lung disease	26 (4%)	25 (4.2%)	1 (1.6%)	0.310
Vaccination Status				
Complete	245 (37.3%)	240 (40.4%)	3 (4.7%)	<0.001
Incomplete	112 (17.0%)	112 (18.9%)	3 (4.7%)	0.006
Unvaccinated	300 (45.8%)	242 (40.7%)	57 (90.5%)	<0.001
Symptoms				
Fever	195 (29.6%)	173 (29.1%)	22 (34.9%)	0.425
Arthralgia	111 (16.9%)	104 (17.5%)	7 (11.1%)	0.150
Myalgia	139 (21.1%)	126 (21.2%)	13 (20.6%)	0.869
Chest Tightness	71 (10.8%)	65 (10.9%)	6 (9.5%)	0.755
Headache	191 (29.1%)	173 (29.1%)	18 (28.6%)	0.927
Anosmia	157 (23.9%)	144 (24.2%)	13 (20.6%)	0.455
Ageusia	131 (19.9%)	121 (20.4%)	10 (15.8%)	0.329
Odynophagia	122 (18.5%)	107 (18.0%)	15 (23.8%)	0.301
Dysgeusia	34 (5.2%)	31 (5.2%)	3 (4.7%)	0.997
Rhinorrhea/cong	151 (22.9%)	141 (23.7%)	10 (15.8%)	0.207
Hyporexia	122 (18.5%)	116 (19.5%)	6 (9.5%)	0.055
Anorexia	36 (5.5%)	31 (5.2%)	5 (7.9%)	0.291
Insomnia	77 (11.7%)	72 (12.1%)	5 (7.9%)	0.358
Cough	187 (28.5%)	174 (29.3%)	13 (20.6%)	0.147
Shortness of Breath	70 (10.6%)	57 (9.6%)	13 (20.6%)	0.003
Expectoration	49 (7.5%)	43 (7.2%)	6 (9.5%)	0.614
Diarrhea	102 (15.5%)	94 (15.8%)	8 (12.7%)	0.972
Malaise	213 (32.4%)	189 (31.8%)	24 (38.1%)	0.297
Nausea	6 (0.9%)	6 (0.9%)	0 (0.0%)	--
Vomiting	2 (0.3%)	2 (0.3%)	0 (0.0%)	--
Initial Covid Stage				
Mild	554 (84.3%)	497 (83.7%)	57 (90.5%)	
Moderate	102 (15.5%)	98 (16.5%)	4 (6.3%)	
Severe	1 (0.15%)	0 (0.0%)	1 (1.6%)	

### 3.1 Primary endpoints

#### 3.1.1 30-day-mortality

**FIGURE 2:**
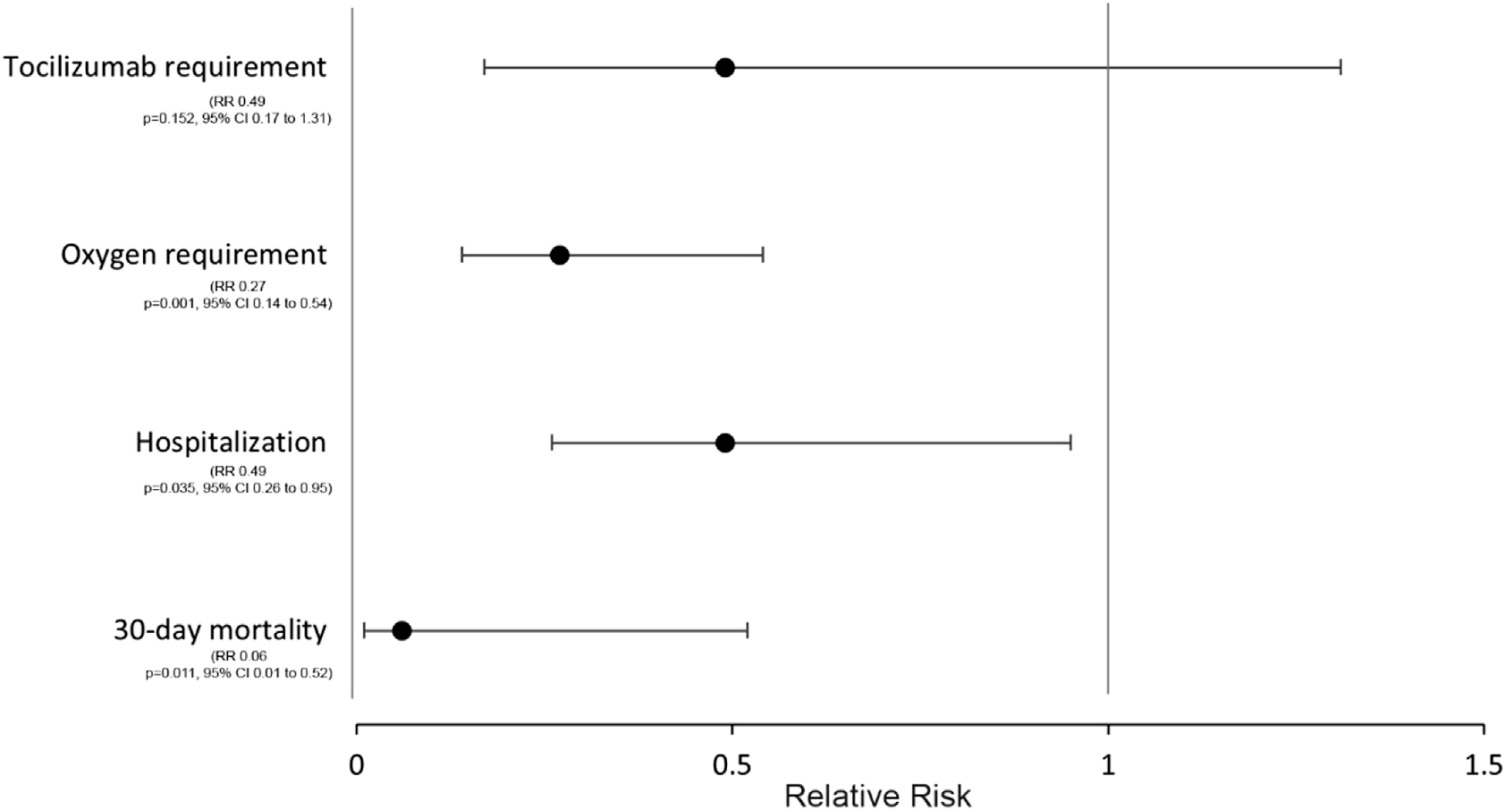
Relative-risk ratio of primary and secondary endpoints in the Fluvoxamine group.

Of the 657 total COVID-19 cases, 5 patients (0.76%) died. Only one death occurred in the fluvoxamine group. Pearson chi-square test showed a significant association between lack of fluvoxamine use and mortality (χ2 = 28.8, *p* < 0.001, Cramer’s V 0.21). Patients who receive fluvoxamine had a significantly lower 30-day mortality risk (RR 0.03, *p* 0.001, CI 0.003–0.233). The patients in the fluvoxamine group had 94% relative risk reduction in the mortality even after stratifying only unvaccinated patients (RR 0.06, *p* 0.011, 95% CI 0.007–0.516) (Table 2; Figure 2). Of note, no statistical difference in 30-day mortality was found between 100 mg vs. 200 mg daily dose of fluvoxamine (*p* = 0.997).

**TABLE 2:** Results: Primary and Secondary Outcomes in unvaccinated patients.

Outcomes	Total no.	Received fluvoxamine	Not- received fluvoxamine	Relative risk ratio, *p* value, 95% CI
	Total	Number (Percent)	Number (Percent)	
Unvaccinated patients	299	242 (80.9%)	57 (19.1%)	
Primary outcomes				
30-day mortality	5	1 (0.4%)	4 (7.0%)	RR 0.06, *p* = 0.011, 95% CI 0.007 to 0.516
Hospitalization	34	23 (9.5%)	11 (19.3%)	RR 0.49, *p* = 0.035, 95% CI 0.26–0.95
Secondary outcomes				
Oxygen requirement	28	15 (6.2%)	13 (22.8%)	RR 0.27, *p* < 0.001, 95% CI 0.14–0.54
Tocilizumab	10	5 (2.0%)	5 (9.0%)	RR 0.49, *p* = 0.152, CI 0.17–1.31
Days in hospital		12 days	7 days	Δ 5 days, *p* 0.08, CI −0.63 to 10.63

#### 3.1.2 Hospitalization

Out of the 657 total patients in the study, 40 (6%) were hospitalized with COVID-19. A total of 5% (30 out of 594) of patients who received fluvoxamine and 15% (10 out of 63) of patients who did not take fluvoxamine were hospitalized. There was a significant difference in hospitalization between the two groups (Pearson χ2 12.07, *p* 0.001). The patients in the fluvoxamine group had 72 percent relative risk reduction in the hospitalization (RR 0.28, *p* < 0.001, 95% CI 0.15–0.53) versus non-fluvoxamine group (Table 2). Of note, there was 51 percent relative risk reduction in hospitalization among fluvoxamine group despite only including unvaccinated patients (RR 0.49, *p* = 0.035, 95% CI 0.26–0.95) (Table 2; Figure 2).

### 3.2 Secondary endpoints

#### 3.2.1 Oxygen requirement

A total of 30 patients required oxygen. Of those patients, 24 were in the hospitalized group and 9 were in the non-hospitalized group. The relative risk ratio of requiring oxygen in patients in fluvoxamine group was 0.16, suggesting relative risk reduction of 84% (*p* < 0.001, CI 0.09–0.31). Of note, there was still a 73 percent reduction in relative risk of requiring oxygen in the fluvoxamine group despite including only unvaccinated patients. (RR 0.27, *p* < 0.001, 95% CI 0.14–0.54) (Table 2; Figure 2).

#### 3.2.2 Tocilizumab requirement

A total of 10 patients required tocilizumab out of the total of 657 patients in the study. The relative risk ratio of requiring tocilizumab in patients who took fluvoxamine was 0.11 (*p* < 0.001, CI 0.03–0.36). However, when stratified by hospitalization, the relative risk ratio of requiring tocilizumab failed to reach significance between two groups (RR 0.49, *p* = 0.152, CI 0.17–1.31) (Table 2; Figure 2).

#### 3.2.3 Days in hospital

The mean hospital stay for hospitalized patients was 8.3 days (median 6 days). There was no significant difference in the mean hospital stay between hospitalized patients from fluvoxamine and non-fluvoxamine groups (10.3 vs. 7.6 days, p0.328) (Table 2).

#### 3.2.4 Critical illness

Out of the 657 total cases, 624 (95.0 percent) remained as mild to moderate COVID-19, while 33 patients (5.0 percent) advanced to severe to critical disease. Median days of disease onset before index visit were three versus four for the fluvoxamine group versus non-fluvoxamine group, respectively. There was no significant difference in median days between both patient groups (*p* = 0.072). However, the fluvoxamine group had a significantly lower number of cases advancing into a severe-to-critical COVID-19 stage than non-fluvoxamine group (Pearson χ2 43.2, *p* < 0.001, Cramer’s V 0.26). The difference remained statistically significant even after including only unvaccinated patients (Pearson χ2 17.7, *p* < 0.001, Cramer’s V 0.24).

#### 3.2.5 Laboratory markers

The mean lymphocytes count on the first follow up visit was significantly higher in the fluvoxamine group (1.72 vs. 1.38, Δ 0.33, *p* < 0.006, 95% CI 0.09–0.58). The mean WBC count on the first follow up visit was significantly different between the non-fluvoxamine and fluvoxamine group (7.8 vs. 5.9, Δ 1.9, *p* < 0.001, CI 0.9–2.9). Mean CRP levels were not statistically different between the non-fluvoxamine and fluvoxamine group (30.3 vs. 19.4, Δ 10.9, *p* 0.216, CI 6.4–28.1). Similarly, mean procalcitonin, d-dimer, and serum ferritin levels were not statistically different between the non-fluvoxamine and the fluvoxamine group (*p* > 0.05).

## 4 Discussion

Multiple mechanisms have been described in which fluvoxamine can prevent deterioration and hospitalization in patients with multiple risk factors for COVID-19 disease progression. SSRIs have been shown to reduce serum serotonin levels by >80% (Duerschmied et al., 2013). Declining levels of serum serotonin reduce platelet aggregation and increase bleeding time (Halperin et al., 2007). In hospitalized COVID-19 patients with acute respiratory distress syndrome (ARDS), there has been described platelet activation hypersensitivity compared to non-COVID-19 ARDS patients (Zaid et al., 2021). While some serotonin is produced in the enterochromaffin cells of the gut, platelets are the main place of storage. Severe COVID-19 patients have increased platelet activation resulting in an amplified release of the serotonin from their storage (Jalali et al., 2021; Martins-Gonçalves et al., 2022). As a consequence, high amounts of plasma serotonin in COVID patients can cause a myriad of problems. Increased serotonin can cause vasoconstriction of smooth muscles in the capillary vessels of the lungs thus making a patient hypoxic (MacLean et al., 2000). Over time, such lack of blood flow in the lungs can cause fibrosis that develops in the later, more severe stages of COVID-19 (Skurikhin et al., 2012). Thirdly, serotonin can heighten platelet aggregation (Almqvist et al., 1984), which can contribute to major cardiovascular events. There is no controversy in the entering third year of the pandemic that these events occur. To counteract this vigorous serotonin release, an intact and healthy pulmonary endothelium is needed to clear the serotonin. This is unlikely to be the case in severe COVID-19 (Dawson et al., 1985; Cloutier et al., 2012; Adnot et al., 2013). Needless to say, anticipating this cascade of events by utilizing a drug like fluvoxamine in the early outpatient makes it appealable on the course of the disease.

There is evidence suggesting that COVID-19 is driven by a constellation of events pointing towards a macrophage activation syndrome. This proinflammatory process recruits’ monocytes that release proinflammatory cytokines (Chen and Subbarao, 2007; Scala and Pacelli, 2020), causing a perpetual state of inflammation. Further detail is beyond the scope of this manuscript; however, extensive reviews have been readily available since the onset of the pandemic. (McGonagle et al., 2020; Merad and Martin, 2020; Iqubal et al., 2021; Marik et al., 2021; McGonagle et al., 2021; Retamozo et al., 2021). In a healthy immune system, Natural Killer (NK) T lymphocytes and Cytotoxic CD8 lymphocytes provide a natural balance in our innate immune system. However, in COVID-19, cytokines present, such as IL-6, can cause T lymphocyte reduction and dysfunction (Mazzoni et al., 2020). In addition, the spike protein lessens the activity of NK T lymphocytes (Bortolotti et al., 2020). The end result of these simultaneous events leads to a transitory lymphopenic state. As a direct consequence, the cytokine release can be incessant and bring with it secondary infections that lead to increased morbidity and mortality of many hospitalized COVID patients.

SSRIs like fluvoxamine also decrease histamine release from mast cells (Ferjan and Erjavec, 1996). Histamine plays a vital role in inflammation, edema, and thrombosis in patients hospitalized by COVID-19. Plasma levels of chymase, a serum marker of mast cell degranulation, were significantly more elevated in hospitalized COVID-19 patients compared to community ambulatory cases (Tan et al., 2021). This elevation signifies the important role mast cells have in the cytokine release phenomena with subsequent exuberant inflammatory response in hospitalized COVID-19 patients. Their role is demonstrated in postmortem COVID-19 patients’ lung biopsies in which activated macrophages were linked to pulmonary edema and thrombosis (Motta Junior et al., 2020). All these mechanisms suggest fluvoxamine has significantly more benefits than its psychotropic effects and can have a critical role in treatment of COVID-19 patients. The sigma-1 receptor chaperone activity is stimulated through the unfolded protein response (UPR), triggered by ER stress, in which the UPR facilitates proper protein folding within the endoplasmic reticulum, preventing cellular stress on the endoplasmic reticulum. If cellular stress on ER is unbearable, it can lead to autophagy and inflammation that can trigger a cytokine storm (Fung et al., 2014).

This most likely is derived from excessive free plasma serotonin levels from virus induced excessive platelet activation, that leads to excessive platelet-fibrin aggregation and formation of microthrombi. This contributes to clinical deterioration, a mechanism which fluvoxamine could block through its serotonin reducing effects on plasma and anti-aggregation effects on platelets. Fluvoxamine has also been shown to play a significant role in modulating inflammation through its agonistic activity on the sigma-1 receptor. Through its chaperone activity, it may protect against mitochondrial damage and endoplasmic reticulum (ER) stress in response to SARS-COV2 infection, as it prevents protein misfolding in the ER because of the ER overloading with virus-encoded proteins (Hayashi et al., 2007; Brimson et al., 2021).

Fluvoxamine, a first-generation selective serotonin reuptake inhibitor (SSRI), showed promising results in several studies in the fight against COVID-19. During early phases of the disease (Lenze et al., 2020; Seftel and Boulware, 2021), two studies showed significant reduction on disease progression, and on more advance life-threatening phases, the study of Calusic et al. demonstrated a positive effect on mortality rate on ICU COVID-19 patients in the treatment group (Calusic et al., 2021). Our study showed similar results in larger cohort of patients seeking early care mostly during the waves of beta, gamma, and delta when vaccines were not available. There was significant relative risk reduction in the hospitalization, mortality, need for oxygen supplementation, and clinical deterioration to a more severe disease stage. Our 30-day mortality finding of 1 death out of 595 (0.16%) is similar to the mortality results of the per-protocol analysis of the TOGETHER trial that showed 1 death in 548 (0.18%) patients that had adequate adherence to Fluvoxamine during the study (Reis et al., 2022).

Fluvoxamine works well with most medications used in the treatment of COVID-19 and there are no significant drug-drug interactions with the exception of anticoagulants. Medications with antiplatelet properties such as fluvoxamine have a tendency to increase the anticoagulant properties of heparin (Samuel and Seifert, 2016; Nochaiwong et al., 2021) Hospitalized COVID-19 patients on both fluvoxamine and heparin will need to be monitored for signs of bleeding and the heparin dosing may need to be reduced. In an outpatient setting, patients on both fluvoxamine and aspirin will need to be counseled that their risk of gastrointestinal bleeding is increased when both medications are used (Dalton et al., 2003). In most cases, the benefits of using both fluvoxamine and an anticoagulant will outweigh the risks.

The discussed anti-inflammatory, hemostatic, anti-viral, and antihistamine properties of fluvoxamine can explain the significant difference of lower hospitalizations and mortality in the treatment group compared to the control group as shown in our study. It is important to mention that we found a lower lymphocyte count in the cohort not taking fluvoxamine. If the lymphocyte count is found higher in the fluvoxamine group in our study, it raises the question of the armamentarium of potential mechanism of actions of fluvoxamine or should it be considered a random finding? This leaves the question open for future investigations, whether fluvoxamine abates cytokine storm and macrophage activation by avoiding lymphopenia?

We do realize our study is not flawless, yet we believe it provides some insight as to the clinical value of a repurposed drug like fluvoxamine. To our knowledge, this is one of the largest real world of study in the use of this drug in COVID-19 disease. Amongst these studies, we stress the novel idea, albeit unexpected critical relevance in the role of lymphopenia or lack thereof in COVID-19 (Akbari et al., 2020; Liu et al., 2020; Sciacchitano et al., 2020; Liu et al., 2021). Although impossible to plot in a perfect biostatistical equation, it should be applauded that this study was performed without any financial backing. These same authors were ahead of their time and heralded in March 2020 that the simultaneous use of corticosteroids and tocilizumab would become the standard of care in severe to critical patients in this disease process a year later in February 2021 (Valerio Pascua et al., 2021). This is the same scientific group that is noticing a reduction in the use of IL-6 inhibitors when fluvoxamine is implemented in earlier stages of the disease.

To close, at the moment of the writing this manuscript, to our awareness this is not only one of the largest studies but also providing mortality similar to the highly funded TOGETHER trial (Cheema et al., 2022). In our opinion fluvoxamine should be implemented as the standard of care in the early outpatient treatment for low-income individuals and nations.

### 4.1 Limitations

Prospective cohort studies, being non-randomized, carry inherent biases related to lack of randomization and confounding variables. However, our study is a “real world” study, involving a fairly large cohort of patients. Although, propensity matching could not be statistically performed due to near absence of vaccinated patients in the non-fluvoxamine group and due to low number of overall events. The univariate analysis showed no significant differences in baseline characteristics between two groups with exception of vaccination status. Therefore, we performed data analysis after excluding vaccinated patients. This makes our data more alike and removes confounding due to vaccination status. This also makes our analysis robust by comparing the effect of fluvoxamine in un-vaccinated patients only who are always at higher risk as compared to their vaccinated peers. Secondly, this study was performed in the outpatient setting. As a result, close hemodynamic monitoring and care of patients was not performed in the hospital. The difference in outcomes with fluvoxamine could be affected by social support and medication compliance. However, our patients received the standard COVID -19 protocol in both arms and followed up with the clinic as per standard study protocol. The lack of ethnic diversity, and the fact that it was conducted in a single hospital during the COVID-19 pandemic are all limiting factors with respect to the study outcomes. The study was conducted at a private hospital, with some patients choosing to be hospitalized even if they did not meet hospitalization criteria. As a result, the hospitalization rate may have been increased. Thirdly, shortness of breath was present in 13/63 (20.6%) cases in non-fluvoxamine group and 57/594 (9.6%) cases in Fluvoxamine group. One possible explanation is that the data is skewed due to the large amount of missing entries, hence limiting our ability to make inferences. Despite all these limitations, there is a counterbalance when using fluvoxamine in an underdeveloped nation as demonstrated in this study. The pandemic has proven to the world that there have been disparities in the distribution of novel therapies directed to SarsCoV2, such as newly developed vaccines and monoclonal antibodies. Given our prior experiences in the distribution of new therapies, it would be naïve to believe that Honduras will be a priority to these big pharmaceutical companies producing these products.

### 4.2 Social and economic impact

Fluvoxamine represents a relatively inexpensive option that is affordable for people who live in extreme poverty in developing countries such as Honduras. Fluvoxamine has already been approved and available in most pharmacies around the world and therefore, it is more accessible, including rural areas. As a result, fluvoxamine will have a bigger impact in the developing nations. The evidence that we presented in this study will have both social and economic impact on the ambulatory management of COVID-19 patients.

## 5 Conclusion

Despite new antiviral medications, such as Molnupiravir and Paxlovid, showing promise in the prevention of disease severity, the production and distribution of these drugs to underserved people in low to middle income countries such Honduras will be limited due to the high demand of these drugs in developed countries. Additionally, the high selling prices of these drugs will limit government investment in these countries. Furthermore, the benefit of fluvoxamine goes beyond its antiviral potential and delivers fundamental benefits by reducing the impact on the mechanisms of disease severity that are associated with death. The study showed a significant mortality and hospitalization benefit among patients who completed treatment with fluvoxamine. These findings were similar to the results of a recent publication, the TOGETHER trial.

## Data Availability

The raw data supporting the conclusion of this article will be made available by the authors, without undue reservation.
